# Supplementary material for: Dynamic control of ferroic domain patterns by thermal quenching
Source: Nat Commun. 2025 Jul 23;16:6802. doi: 10.1038/s41467-025-62158-2 (PMC12287398; doi:10.1038/s41467-025-62158-2)
Supplement: Supplementary file 1 — Supplementary Information [file 41467_2025_62158_MOESM1_ESM.pdf]

Supporting information: Dynamic control of ferroic domain patterns by thermal quenching

Jan Gerrit Horstmann[1*], Ehsan Hassanpour[1], Aaron Merlin Müller[1], Yannik Zemp[1], Thomas Lottermoser[1], Yusuke Tokunaga[2], Yasujiro Taguchi[3], Yoshinori Tokura[3,4], Mads C. Weber[5], Manfred Fiebig[1]

[1]Department of Materials, ETH Zurich, Vladimir-Prelog-Weg 4, Zurich, 8093, Switzerland.
[2]Department of Advanced Materials Science, The University of Tokyo, Chiba, 277-8561, Japan.
[3]RIKEN Center for Emergent Matter Science (CEMS), Saitama, 351-0198, Japan.
[4]Department of Applied Physics and Tokyo College, The University of Tokyo, Tokyo, 113-8656, Japan.
[5]Institut des Molécules et Matériaux du Mans, UMR 6283 CNRS, Le Mans Université, Le Mans, 72085, France.

*Corresponding author(s). E-mail(s): jan-gerrit.horstmann@mat.ethz.ch;

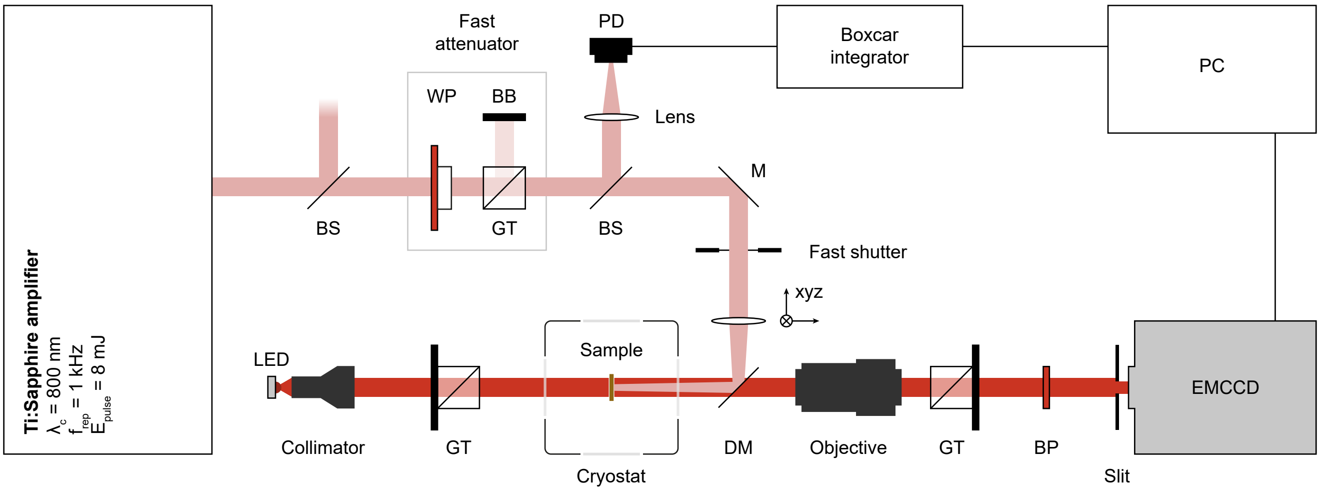

Fig. S1: Experimental setup. To image the dynamics of the wFM order in DTFO during rapid thermal quenches, the output of a LED is collimated and polarized by a Glan-Taylor prism (GT). The probe beam is guided into the optical cryostat, transmitted through the sample, and collected by a camera objective. The image contrast is generated by adjusting a second Glan-Taylor prism (GT) to filter the rotated polarization component. The light passes a band pass filter (BP) and passes an adjustable slit array used to mask selected parts of the detector (EMCCD) for imaging at enhanced frame rates. Sample excitation is realized by focusing the output of a Ti:Sapphire amplifier onto the sample by a plano-convex lens. The beam can be attenuated with high precision and speed using the combination of a half-wave plate (WP) and a Glan-Taylor prism (GT). BB, beam block. A glass slide (BS) is used to redirect a small fraction of the attenuated beam onto a photodiode (PD) to record a reference signal proportional to the sample excitation during heating, annealing and quenching. A fast shutter can be used to create even higher gradients in the time-dependent optical excitation profile. The pump beam is guided onto the back-side of the sample via a dichroic mirror (DM), which is highly reflective in the wavelength range of the pump, and highly transmissive at the probe wavelength of 650 nm.

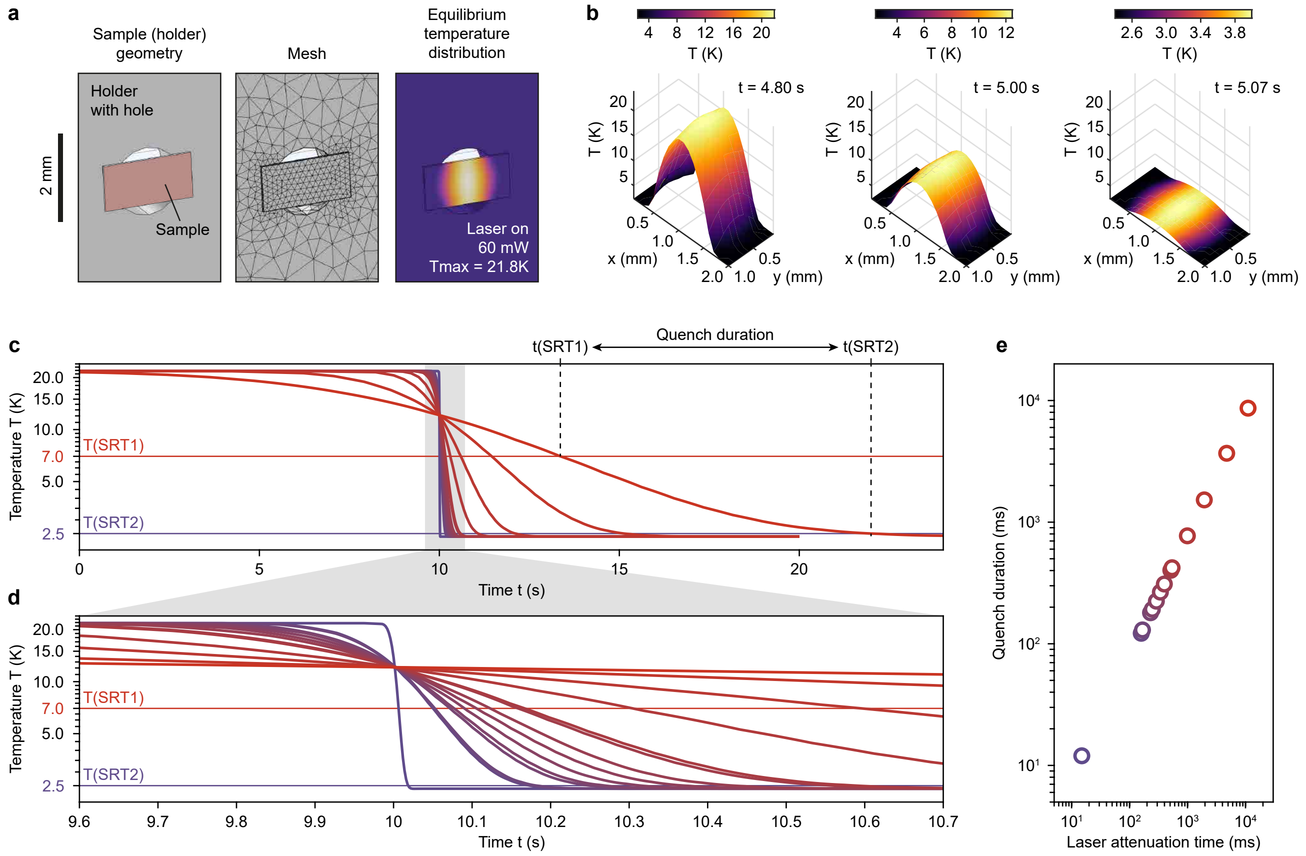

Fig. S2: Details of the COMSOL® simulations. a, Sample geometry (left), mesh applied for finite element calculations (middle), and equilibrium temperature distribution at the highest laser power of 60 mW (right). **b**, Two-dimensional temperature distribution on the sample at three selected times during a simulated thermal quench with a laser attenuation time of 159 ms. Note that the error function governing the time-dependent heating power is centered around $t = 10$ s. **c**, Temperature at the center of the sample or laser spot, respectively, as a function of time across thermal quenches with laser attenuation times used in the experiments. Horizontal red and violet lines mark the temperatures of SRT1 and SRT2. The quench duration Δt_q is determined by evaluating t_{SRT1} and t_{SRT1} at $T = T_{\mathrm{SRT1}}$ and $T = T_{\mathrm{SRT1}}$. **d**, Time-dependent sample temperatures in a smaller time interval around $t = 10$ s (same dataset as in **c**). **e**, Calculated quench duration as a function of the laser attenuation time measured by the reference photo-diode. The linear relation between laser attenuation time and Δt_q over several orders of magnitude evidences that the sample temperature quasi-instantaneously follows changes in laser power on the ms time scales relevant for our experiments.

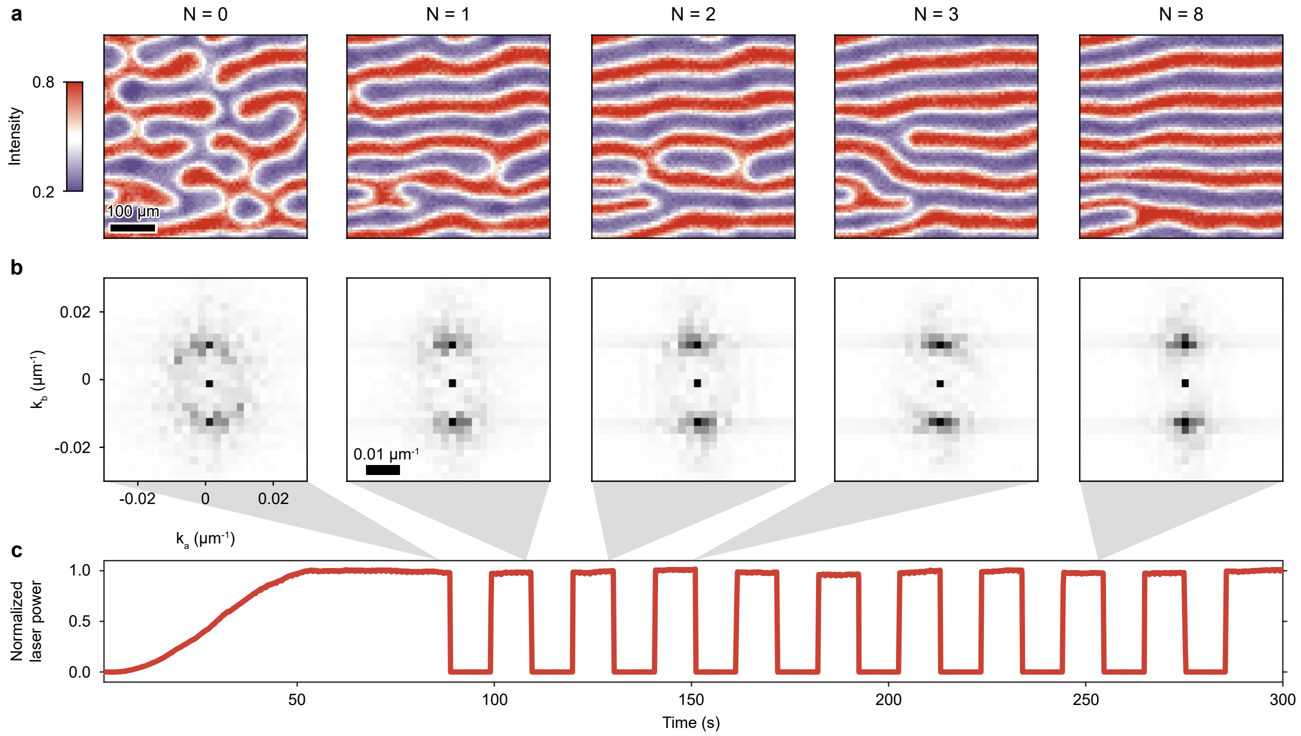

Fig. S3: Back and forth quenches between LT- and HT-F_z phases. a, Faraday images of the sample in the HT-F_z phase recorded after N fast laser-assisted quenches into the LT-F_z phase and back. With increasing N, the initial maze-domain pattern is transformed step-by-step into a stripe-domain pattern due to the repeated passage of the intermediate F_x phase. Because of the preservation of the domain pattern between the two phases during quenches, the creation or annihilation of individual topological defects can be observed. The quench-induced stripe pattern in the HT-F_z phase is stable for many hours. Maze domains can only be regained by heating the sample up to $\sim 100\,\mathrm{K}$ and slowly cooling down again. **b**, Two-dimensional Fourier transforms of the respective images in **a** highlight the stepwise loss of isotropy of the domain pattern due to stripe formation. **c**, Time-dependent laser power on the sample during the successive heating/cooling cycles. Based on these results and the data presented in Fig. 2, we conclude that both the HT-F_z and the LT-F_z phases exhibit metastable maze domain and stripe domain configurations. Whereas maze domains are energetically favorable in the HT-F_z, stripe domains likely represent the lower-energy configuration in the LT-F_z phase. Consequently, stripe (maze) domains represent a metastable configuration in the HT-F_z (LT-F_z) phase.

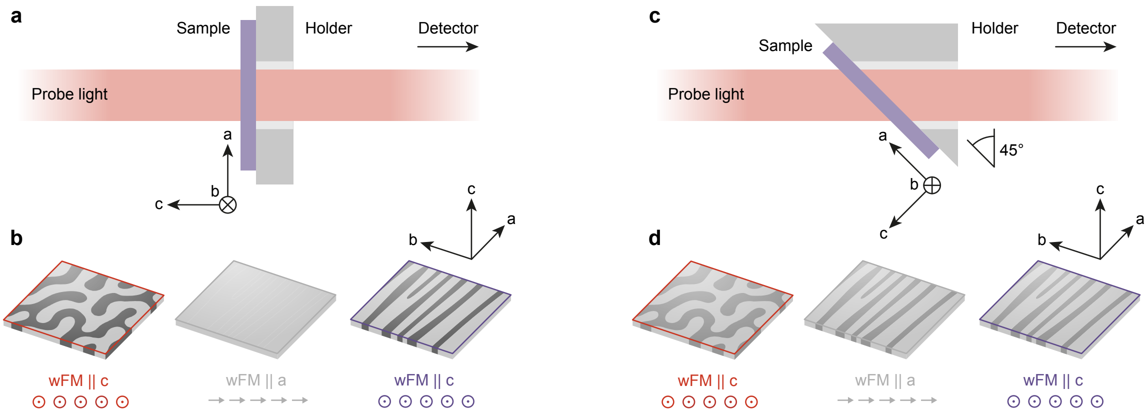

Fig. S4: Measurement geometries. **a**, Sketch of the sample orientation for the measurements presented in Fig. 2 and Fig. 3. **b**, Schematic depiction of the image contrast in F_z and F_x phases for vertical sample orientation. **c**, Sketch of the sample orientation for the measurements presented in Fig. 1 and Fig. 4. **d**, Schematic depiction of the image contrast in F_z and F_x phases for tilted samples.

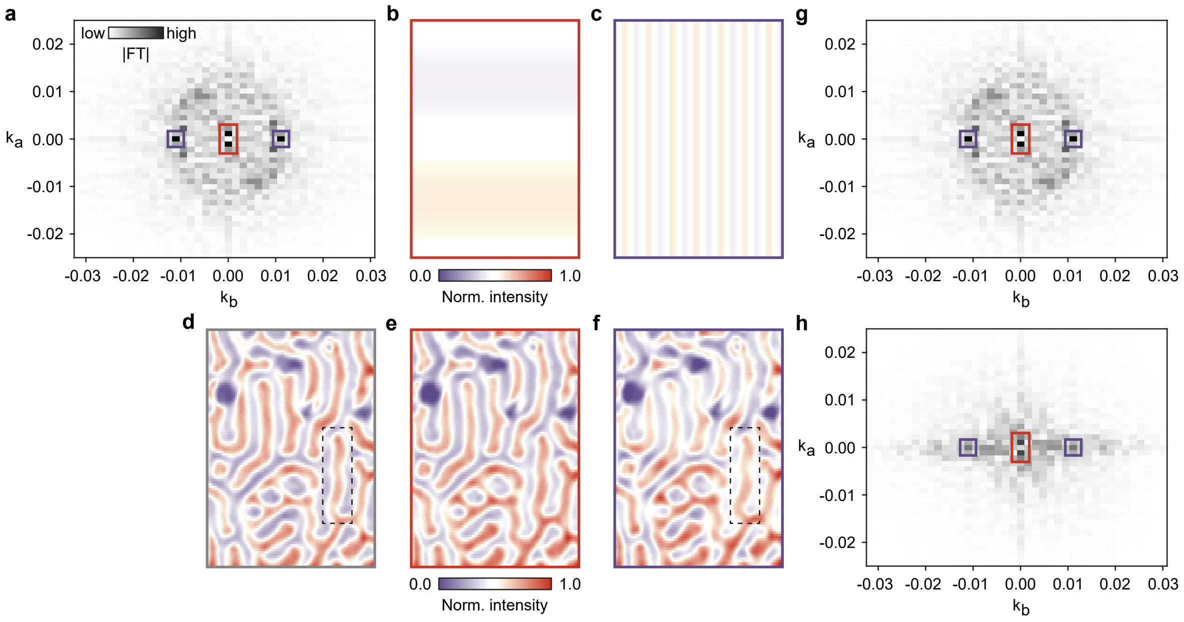

Fig. S5: Supplement to the Fourier analysis in Fig. 4. **a**, 2D Fourier transform of the initial HT-F_z domain pattern taken from the dataset underlying Fig. **??**b-d. Red and violet rectangles highlight two types on Fourier components dominating the 2D FT around $k_\mathrm{a} = 0$ (see also the prominent feature at $k_\mathrm{a} = 0$ before $t = 0\,\mathrm{ms}$ in Fig. 4b). **b**, Real space pattern yielded by an inverse FT of the two prominent Fourier components (red) in **a**. These features in the FT correspond to a vertical intensity gradient across the entire image caused by a slightly inhomogeneous illumination of the sample. **c**, Real space pattern yielded by an inverse FT of the two prominent Fourier components (violet) in **a**. We associate this feature with the general anisotropy of the system, which is present in all phases, including the HT-F_z phase. In the case of the F_x and LT-F_z phases, this anisotropy leads to the formation of stripes along the a-direction. For the HT-F_z phase, other interactions dominate domain formation. However, the characteristic maze domains of the HT-F_z do exhibit a slight tendency to align along the a-direction resulting in peaks at $(k_a = 0, k_b = \pm 0.011\,\mu\mathrm{m}^{-1})$ in the FT. **d**, Original domain image. **e**, Domain image yielded by an inverse FT of **a** after filtering out the components highlighted in red. Notice the smaller intensity gradient along the vertical direction in **e** compared to **d**. **f**, Domain image yielded by an inverse FT of **a** after filtering out the components highlighted in violet. Compare the slightly larger modulation of domains along b highlighted by the black dashed rectangles in **d** and **f**. Overall, the highlighted features in Fourier space do not dominate the domain pattern and are therefore not relevant for the analysis presented in the main text. **g-h**, FTs of domain images before (**g**) and shortly after (**h**) SRT1. Whereas features associated with the intensity gradient (red) are present throughout the entire duration of the measurement, features corresponding to the systems anisotropy (violet) are transiently suppressed following SRT1.

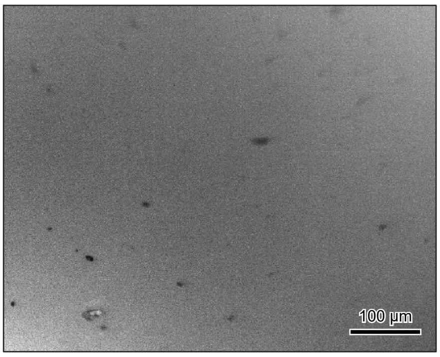

Fig. S6: Room-temperature domain pattern in a-cut DTFO. Faraday image of an a-cut sample rotated by 45 ° around the *b*- and *c*-axes. No weak ferromagnetic (wFM) stripe domains are observed, indicating a single-domain state. This supports our hypothesis that the wFM stripe pattern in the F_x phase originates from anisotropy along the *a*-axis, induced by paramagnetically oriented rare-earth moments at temperatures well below room temperature.

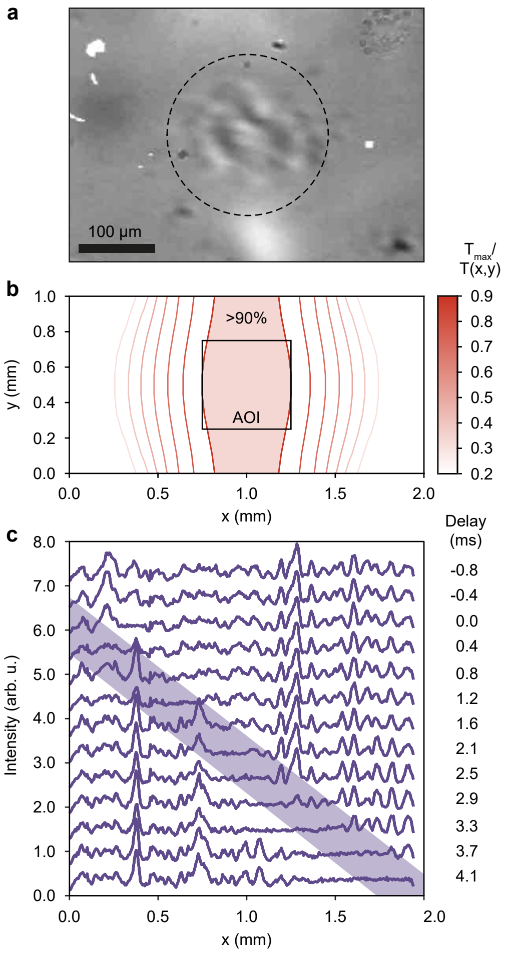

Fig. S7: Experimental and computational details. **a**, Exemplary image of laser-illuminated DTFO. Note that in actual experiments, the laser spot diameter is significantly larger (laser spot diameter $d = 500\,\mu$m). Whereas the outer part of the sample is heated to the F_x phase (see loss of domain contrast), the inner part close to the center of the laser spot is driven into the HT-F_z phase (see fluctuating maze domains). **b**, Contour plot of the sample temperature under constant laser illumination. The temperature values $T(x, y)$ are normalized by the maximum temperature at the center of the laser spot. Within the rectangular area of interest (AOI) imaged in our experiments, the temperature varies by less than 10% relative to the maximum value at the center. **c**, Measured propagation of the phase front between LT-F_z and F_x phases for a localized optical excitation of the sample close to $x = 0$. Analyzing the changes in the domain structure (broad violet line, guide to the eye) as a function of the delay with respect to the step-like excitation yields a phase front velocity of $500\,\mu\mathrm{m\,ms}^{-1}$. This highlights that the sample responds quasi-instantaneously to changes in laser power across the entire probed region on the ms time scale relevant for our experiments.
