# Supplementary material for: Dynamic control of ferroic domain patterns by thermal quenching
Source: Nat Commun. 2025 Jul 23;16:6802. doi: 10.1038/s41467-025-62158-2 (PMC12287398; doi:10.1038/s41467-025-62158-2)
Supplement: Supplementary file 2 — Reporting Summary [file 41467_2025_62158_MOESM2_ESM.pdf]

natureresearch

Corresponding author(s): Jan Gerrit Horstmann

Lasing Reporting Summary

Nature Research wishes to improve the reproducibility of the work that we publish. This form is intended for publication with all accepted papers reporting claims of lasing and provides structure for consistency and transparency in reporting. Some list items might not apply to an individual manuscript, but all fields must be completed for clarity.

For further information on Nature Research policies, including our data availability policy, see Authors & Referees.

Experimental design

Please check: are the following details reported in the manuscript?

1. Threshold

Plots of device output power versus pump power over a wide range of values indicating a clear threshold

- [ ] Yes
- [x] No

Commercial laser system (Coherent Legend Elite Duo) has been used

2. Linewidth narrowing

Plots of spectral power density for the emission at pump powers below, around, and above the lasing threshold, indicating a clear linewidth narrowing at threshold

- [ ] Yes
- [x] No

Commercial laser system (Coherent Legend Elite Duo) has been used

Resolution of the spectrometer used to make spectral measurements

- [ ] Yes
- [x] No

Commercial laser system (Coherent Legend Elite Duo) has been used

3. Coherent emission

Measurements of the coherence and/or polarization of the emission

- [ ] Yes
- [x] No

Commercial laser system (Coherent Legend Elite Duo) has been used

4. Beam spatial profile

Image and/or measurement of the spatial shape and profile of the emission, showing a well-defined beam above threshold

- [ ] Yes
- [x] No

Commercial laser system (Coherent Legend Elite Duo) has been used

5. Operating conditions

Description of the laser and pumping conditions
Continuous-wave, pulsed, temperature of operation

- [ ] Yes
- [x] No

Commercial laser system (Coherent Legend Elite Duo) has been used

Threshold values provided as density values (e.g. W cm^{-2} or J cm^{-2}) taking into account the area of the device

- [ ] Yes
- [x] No

Commercial laser system (Coherent Legend Elite Duo) has been used

6. Alternative explanations

Reasoning as to why alternative explanations have been ruled out as responsible for the emission characteristics
e.g. amplified spontaneous, directional scattering; modification of fluorescence spectrum by the cavity

- [ ] Yes
- [x] No

Commercial laser system (Coherent Legend Elite Duo) has been used

7. Theoretical analysis

Theoretical analysis that ensures that the experimental values measured are realistic and reasonable
e.g. laser threshold, linewidth, cavity gain-loss, efficiency

- [ ] Yes
- [x] No

Commercial laser system (Coherent Legend Elite Duo) has been used

8. Statistics

Number of devices fabricated and tested

- [ ] Yes
- [x] No

Commercial laser system (Coherent Legend Elite Duo) has been used

Statistical analysis of the device performance and lifetime (time to failure)

- [ ] Yes
- [x] No

Commercial laser system (Coherent Legend Elite Duo) has been used

March 2018
